# Construct validity of the Post-COVID-19 Functional Status Scale in adult subjects with COVID-19

**DOI:** 10.1186/s12955-021-01691-2

**Published:** 2021-02-03

**Authors:** Felipe V. C. Machado, Roy Meys, Jeannet M. Delbressine, Anouk W. Vaes, Yvonne M. J. Goërtz, Maarten van Herck, Sarah Houben-Wilke, Gudula J. A. M. Boon, Stefano Barco, Chris Burtin, Alex van ’t Hul, Rein Posthuma, Frits M. E. Franssen, Yvonne Spies, Herman Vijlbrief, Fabio Pitta, Spencer A. Rezek, Daisy J. A. Janssen, Bob Siegerink, Frederikus A. Klok, Martijn A. Spruit

**Affiliations:** 1 Department of Research and Development, CIRO+, PO Box 4080, 6080 AB Horn, Haelen, The Netherlands; 2 NUTRIM School of Nutrition and Translational Research in Metabolism, Faculty of Health, Medicine and Life Sciences, Maastricht University, Maastricht, The Netherlands; 3 Department of Respiratory Medicine, Maastricht University Medical Center (MUMC+), Maastricht, The Netherlands; 4 Laboratory of Research in Respiratory Physiotherapy (LFIP), Department of Physiotherapy, State University of Londrina (UEL), Londrina, Brazil; 5 REVAL Rehabilitation Research Center, BIOMED Biomedical Research Institute, Faculty of Rehabilitation Sciences, Hasselt University, Diepenbeek, Belgium; 6 Department of Medicine – Thrombosis and Hemostasis, Leiden University Medical Center, Leiden, The Netherlands; 7 Center for Thrombosis and Haemostasis (CTH), University Medical Center of the Johannes Gutenberg University Mainz, Mainz, Germany; 8 Clinic of Angiology, University Hospital Zurich, Zurich, Switzerland; 9 Department of Respiratory Diseases, Radboud Institute for Health Sciences, Radboud University Medical Center, Nijmegen, The Netherlands; 10 Lung Foundation Netherlands, Amersfoort, The Netherlands; 11 Institute of Therapies and Rehabilitation, Kantonsspital Winterthur, Winterthur, Switzerland; 12 Department of Health Services Research, Care and Public Health Research Institute, Faculty of Health, Medicine and Life Sciences, Maastricht University, Maastricht, The Netherlands; 13 Center for Stroke Research Berlin, Charité Universitätsmedizin Berlin, Berlin, Germany; 14 Department of Clinical Epidemiology, Leiden University Medical Center, Leiden, The Netherlands

**Keywords:** SARS-CoV-2, Functional status, Symptoms, Quality of life

## Abstract

**Background:**

An increasing number of subjects are recovering from COVID-19, raising the need for tools to adequately assess the course of the disease and its impact on functional status. We aimed to assess the construct validity of the Post-COVID-19 Functional Status (PCFS) Scale among adult subjects with confirmed and presumed COVID-19.

**Methods:**

Adult subjects with confirmed and presumed COVID-19, who were members of an online panel and two Facebook groups for subjects with COVID-19 with persistent symptoms, completed an online survey after the onset of infection-related symptoms. The number and intensity of symptoms were evaluated with the Utrecht Symptom Diary, health-related quality of life (HrQoL) with the 5-level EQ-5D questionnaire, impairment in work and activities with the Work Productivity and Activity Impairment questionnaire and functional status with the PCFS Scale.

**Results:**

1939 subjects were included in the analyses (85% women, 95% non-hospitalized during infection) about 3 months after the onset of infection-related symptoms. Subjects classified as experiencing ‘slight’, ‘moderate’ and ‘severe’ functional limitations presented a gradual increase in the number/intensity of symptoms, reduction of HrQoL and impairment in work and usual activities. No differences were found regarding the number and intensity of symptoms, HrQoL and impairment in work and usual activities between subjects classified as experiencing ‘negligible’ and ‘no’ functional limitations. We found weak-to-strong statistical associations between functional status and all domains of HrQoL (r: 0.233–0.661). Notably, the strongest association found was with the ‘usual activities’ domain of the 5-level EQ-5D questionnaire.

**Conclusion:**

We demonstrated the construct validity of the PCFS Scale in highly-symptomatic adult subjects with confirmed and presumed COVID-19, 3 months after the onset of symptoms.

## Background

As of January, 12th 2021, more than 88.3 million confirmed cases of the corona virus disease 2019 (COVID-19) causing approximately 1.900.000 deaths were reported globally [1]. Nevertheless, the total number of subjects with COVID-19 is probably higher due to insufficient testing capacity and/or difficulties in identifying mild cases [2–4]. Previous severe acute respiratory syndrome (SARS) outbreaks showed to affect the survivors’ lung function, exercise capacity, health-related quality of life (HrQoL), mental health, and lead to increased symptoms of fatigue and dyspnea from 6 months to 2 years after symptom onset [5–7]. Due to the increasing number of subjects recovering from the infection of severe acute respiratory syndrome coronavirus 2 (SARS-CoV-2), the need for tools to measure and monitor the course of the disease and its impact on functional status has been raised as essential [8].

Klok and colleagues proposed the “Post-COVID-19 Functional Status (PCFS) Scale” to be used as a patient-reported outcome measure to evaluate the consequences of COVID-19 and their effect on functional status [8]. The PCFS Scale can be used both at the time of hospital discharge, and to monitor functional status post discharge [8]. The scale was designed to cover the entire range of functional limitations from: grade 0, “No functional limitations” to grade 4, “Severe functional limitations” and grade 5, “Death”. Notably, the scale was derived for measuring functional outcomes after venous thromboembolism (VTE), by use of a Delphi method among international VTE experts and patient focus groups [9, 10]. It was found to have good to excellent interobserver agreement between self-reported scale assessment and a structured interview by a trained physician [10]. Since COVID-19 represents an acute cardiopulmonary disease, and has been shown to be frequently complicated by VTE [11], the scale was assumed to be relevant and useful in the clinical course of COVID-19 too [8]. The scale and supporting information such as a manual and various translations are freely accessible via https://osf.io/qgpdv/ (CC-BY 4.0). To date, however, no study has investigated any measurement property of the PCFS Scale in post-COVID-19 subjects.

The aim of the present study was, therefore, to assess the construct validity of the PCFS Scale among adult subjects with confirmed and presumed COVID-19 by testing the hypothesis that this simple tool is associated with the number and intensity of symptoms, HrQoL and impairment in work and usual activities due to health.

## Methods

### Study design, setting and participants

This cross-sectional, survey study was conducted in the Netherlands and Flanders (Belgium) between June 4th and June 11th 2020. Members of two Facebook groups for COVID-19 subjects with persistent complaints [12, 13] and subjects who registered at a website of the Lung Foundation Netherlands (www.coronalongplein.nl) received a web link with an invitation to fill out questionnaires.

Subjects were asked one-off to provide information regarding anthropometric/sociodemographic characteristics and medical history (gender, age, height, body weight, date of onset of symptoms, hospitalization at the time of the infection, type of COVID-19 diagnosis, number of pre-existing comorbidities, marital and smoking status). Subjects were stratified into four groups according to the type of COVID-19 diagnosis: (1) hospitalized with confirmed COVID-19 (regular ward, no admission to intensive care unit (ICU); (2) non-hospitalized with confirmed COVID-19 (based on reverse transcription polymerase chain reaction (RT-PCR) test and/or computed tomography (CT) scan of the thorax); (3) non-hospitalized with symptom-based diagnosis of COVID-19 (established by a doctor, no formal COVID-19 testing); and (4) non-hospitalized with presumed COVID-19 (no formal diagnosis at the time of the presumed infection).

The medical ethics committee of Maastricht University stated that the Medical Research Involving Human Subjects Act (WMO) does not apply for this study and that an official approval of this study by the committee was not required (METC2020-1978). The medical ethics committee of Hasselt University formally judged and also approved the study (MEC2020/041). All subjects gave digital informed consent at the start of the survey. Data from this study on persistent symptoms and care dependency have been published before [14–16].

### Construct validity

The present study adopted the definition of one of the three domains of validity (construct validity) described in the COSMIN study—which aimed to clarify and standardize terminology and definitions of measurement properties that are relevant and should be evaluated for health-related patient-reported outcomes (HR-PRO) [17]. Construct validity is defined as the degree to which the scores of a HR-PRO instrument are consistent with hypotheses (for instance with regard to relationships to scores of other instruments) based on the assumption that the HR-PRO instrument validly measures the construct to be measured [17]. For this reason, we opted to investigate the construct validity of the PCFS Scale by testing whether the PCFS Scale is a simple tool that can be used for measuring the impact of symptoms on the functional status of subjects. Our hypothesis is that the PCFS Scale is related with instruments used to assess the number and intensity of symptoms as well as with instruments which assess the impairment in HrQoL and in work and usual activities. In order to test this hypothesis, we explored the level of impairment in these outcomes (see below) after stratifying subjects according to the PCFS Scale.

### Measures

To assess the level of impairment in functional status subjects were asked to fill in the PCFS Scale [8]. The PCFS Scale stratification is composed of five scale grades: grade 0 (No functional limitations); grade 1 (Negligible functional limitations); grade 2 (Slight functional limitations); grade 3 (Moderate functional limitations) and grade 4 (Severe functional limitations). The final scale grade 5 ‘death’, which is required to be able to use the scale as outcome measure in clinical trials, was left out for this self-administered questionnaire. Additionally, the following questionnaires were completed: the Utrecht Symptom Diary (USD), an adapted Dutch version of the Edmonton Symptom Assessment System [18, 19], to assess symptom intensity ranging from 0 (no symptom) to 10 points (worst possible); the 5-level version of the EQ-5D questionnaire (EQ-5D-5L) to assess HrQoL [20]. This questionnaire is composed by five domains (mobility, self-care, usual activities, pain/discomfort and anxiety/depression) and a visual analogue scale (VAS) ranging from 0 (the worst imaginable health) to 100 points (the best imaginable health). From the scores of each domain, an index can be calculated (ranging from − 0.329 to 1) with higher scores representing a better HrQoL [21]; and the Work Productivity and Activity Impairment (WPAI) questionnaire from which four main outcomes can be generated and expressed in percentages: (1) percent work time missed due to health (absenteeism); (2) percent impairment while working due to health (presenteeism); (3) percent overall work impairment due to health; (4) percent activity impairment due to health. The recall period is 7 days and higher scores represent more impairment in outcomes, the questionnaire has shown to be valid and reproducible and has been extensively used in different common chronic diseases [22, 23].

### Statistical analyses

Continuous variables are presented as mean and standard deviation or median and interquartile range, as appropriate. Categorical variables are presented as absolute and relative frequency. The comparisons of continuous variables between subjects with different levels of impairment in functional status were performed with Kruskal–Wallis test, as the dependent variables were not normally distributed for each group of the PCFS Scale. The comparisons of categorical variables between subjects with different level of impairment in functional status were performed with a Chi-square test of independence or Mantel–Haenszel test of trend, as appropriate. The tests were followed by Bonferroni adjustments for multiple comparisons. An ordinal logistic regression was performed to determine which of the baseline characteristics were associated with a higher odd of being in a higher grade of the PCFS Scale. Statistics and visualization were performed using SPSS (version 25, IBM Corporation, Armonk, NY, USA) and GraphPad Prism 9.0 (GraphPad Software Inc., USA). A priori, the level of significance was set at *P* < 0.05.

## Results

### General characteristics

From the initial 2159 respondents, 15 were excluded because of ICU admission during the infection, 22 were excluded because of reported onset of symptoms before January 1, 2020 or within the previous 21 days, and 183 were excluded due to incomplete surveys. Therefore, a total of 1939 subjects (85% women, 46 ± 11 years, body mass index (BMI): 25.2[22.6–28.8]kg/m^2^) were included in the analyses. The general characteristics of these subjects are presented in Table 1. The time from onset of symptoms to the day of the participation in the study was on average 79 ± 17 days. The majority of the subjects was diagnosed based on symptoms (42%), whereas 17% were diagnosed based on CT/RT-PCR and 36% had no formal COVID-19 diagnosis. Five percent of the subjects were hospitalized with confirmed COVID-19. In addition, the majority of subjects were married or living together (71%), reported no previous comorbidities (61%), and had no previous smoking habits (82%). Most of the subjects reported moderate-to-slight functional limitations according to the PCFS Scale (85%) while only 3% of the subjects reported to currently have no limitations in daily life.

**Table 1:** Baseline characteristics of the subjects with COVID-19 stratified according to the level of impairment in functional status

Variables	Total sample	Post-COVID-19 Functional Status Scale				
		Grade = 0	Grade = 1	Grade = 2	Grade = 3	Grade = 4
	(n = 1939)	(n = 58, 3%)	(n = 157, 8%)	(n = 643, 33%)	(n = 1011, 52%)	(n = 70, 4%)
*General*						
Women, n (%)	1652 (85)	46 (79)	118 (75)	538 (84)	890 (88)^b^	60 (86)
Age (years)	46 ± 11	51 ± 12	47 ± 11	46 ± 11^a^	46 ± 10^a^	45 ± 12^a^
BMI (kg/m^2^)	25.2 [22.6–28.8]	26.1 [24.3–29.1]	25.6 [23.1–28.3]	25.2 [22.7–28.7]	25.2 [22.5–29.3]	22.5 [20.5–26.7]^a,b,c,d^
Underweight (BMI < 18.5), n (%)	27 (1)	0 (0)	2 (1)	4 (0.6)	18 (2)	3 (4)^c^
Normal weight (BMI: 18.5–24.9), n (%)	901 (47)	21 (36)	67 (43)	301 (47)	468 (46)	44 (63)^a,b^
Overweight (BMI: 25.0–29.9), n (%)	621 (32)	25 (43)	66 (42)	209 (33)	307 (31)^b^	14 (20)^a,b^
Obesity (BMI ≥ 30), n (%)	389 (20)	12 (21)	22 (14)	128 (20)	218 (22)	9 (13)
Time since first symptoms (days)	79 ± 17	77 ± 21	82 ± 19	79 ± 17	79 ± 16	83 ± 15
Number of reported symptoms, (n)	11 ± 3	7 ± 4	9 ± 4	10 ± 3^a,b^	11 ± 3^a,b,c^	12 ± 3^a,b,c,d^
*COVID-19 Diagnosis*						
Hospitalized, test-based diagnosis, n (%)	102 (5)	9 (15)	6 (4)^a^	31 (5)^a^	52 (5)^a^	4 (6)
Non-hospitalized, test-based diagnosis, n (%)	319 (17)	17 (29)	30 (19)	120 (19)	144 (14)^a^	8 (11)
Non-hospitalized, symptom-based diagnosis, n (%)	820 (42)	14 (24)	43 (27)	253 (39)	473 (47)^a,b,c^	37 (53)^a,b,c^
Non-hospitalized, no formal diagnosis, n (%)	698 (36)	18 (31)	78 (50)	239 (37)^b^	342 (34)^b^	21 (30)
*Self-reported pre-existing comorbidities*						
None, n (%)	1182 (61)	38 (65)	98 (62)	406 (63)	609 (60)	31 (44)^c^
1 comorbidity, n (%)	499 (26)	15 (26)	47 (30)	161 (25)	259 (26)	17 (24)
≥ 2 comorbidities, n (%)	258 (13)	5 (9)	12 (8)	76 (12)	143 (14)	22 (31)^a,b,c,d^
*Marital Status*						
Alone, n (%)	404 (21)	14 (24)	26 (17)	126 (20)	211 (21)	27 (39)^b,c,d^
Married/Living Together, n (%)	1381 (71)	42 (72)	120 (76)	469 (73)	712 (70)	38 (54)^b,c,d^
Divorced, n (%)	130 (7)	2 (3)	8 (5)	41 (6)	74 (7)	5 (7)
Widow(er), n (%)	24 (1)	0 (0)	3 (2)	7 (1)	14 (1)	0 (0)
*Smoking Status*						
Current smoker, n (%)	119 (6)	3 (5)	4 (2)	39 (6)	68 (7)	5 (7)
Stopped smoking after COVID-19, %, n (%)	226 (12)	8 (14)	23 (15)	78 (12)	108 (11)	9 (13)
Never smoked, n (%)	1594 (82)	47 (81)	130 (83)	526 (82)	835 (83)	56 (80)

*COVID-19* corona virus disease 2019, *BMI* body mass index

^a^*P* < 0.05 compared with Grade 0; ^b^*P* < 0.05 *compared with Grade 1;* ^c^*P* < 0.05* compared with Grade 2; *^d^*P* < 0.05 *compared with Grade 3*

### Stratification for the grade on the PCFS Scale

The gender distribution, type of COVID-19 diagnosis, presence of self-reported pre-existing comorbidities and marital status were different among the PCFS Scale categories. Interestingly, no differences in the proportion of smoking status were presented (Table 1). Additional file 1: Fig. 1 displays the odds of being in a higher grade of the PCFS Scale according to gender, BMI category, type of COVID-19 diagnosis, marital status, presence of self-reported pre-existing comorbidities and smoking status. Additional file 1: Table 1 displays the prevalence of each specific pre-existing comorbidities according to the different groups of the PCFS Scale grades.

Subjects with no functional limitations were older compared to subjects presenting slight, moderate and severe functional limitations. Subjects with severe functional limitations (Grade 4 on the PCFS Scale) presented lower BMI compared to all other groups (Table 1). Notably, other factors associated with poorer functional status were marital status (prevalence of category ‘alone’ highest in Grade 4) and presence of comorbidities (prevalence of ‘≥ 2 comorbidities’ highest in Grade 4). Interestingly, subjects with severe functional limitations also had the highest prevalence of a ‘symptom-based’ COVID-19 diagnosis.

**Fig. 1:**
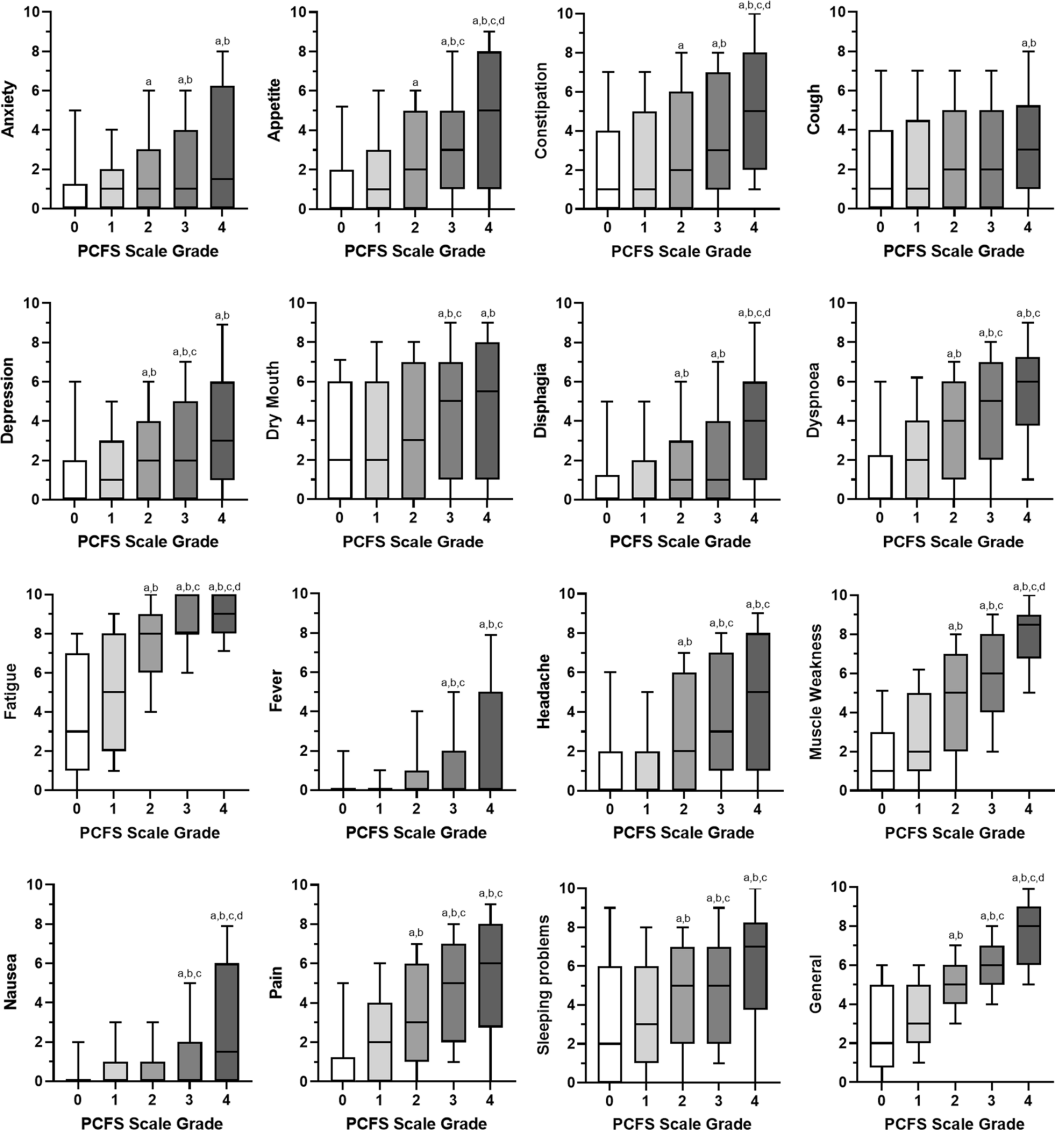
Comparisons of symptoms intensity between subjects with COVID-19 stratified according to the level of impairment in functional status. Figure displays the median as central line, interquartile range as the limits of the box and 10 and 90 percentiles as whiskers. **a** *P* < 0.05 compared with Grade 0; **b** *P* < 0.05* compared with Grade 1;*** c** *P* < 0.05 *compared with Grade 2;*** d** *P* < *0.05 compared with Grade 3*

The most intense symptoms reported were fatigue, muscle weakness and sleeping problems while the least were fever, nausea and dysphagia. Subjects classified as experiencing slight, moderate and severe functional limitations presented a gradual increase in the number and intensity of symptoms (Table 1 and Fig. 1). No differences were found between subjects with negligible functional limitations and no functional limitations concerning the number and intensity of symptoms (Fig. 1).

**Fig. 2:**
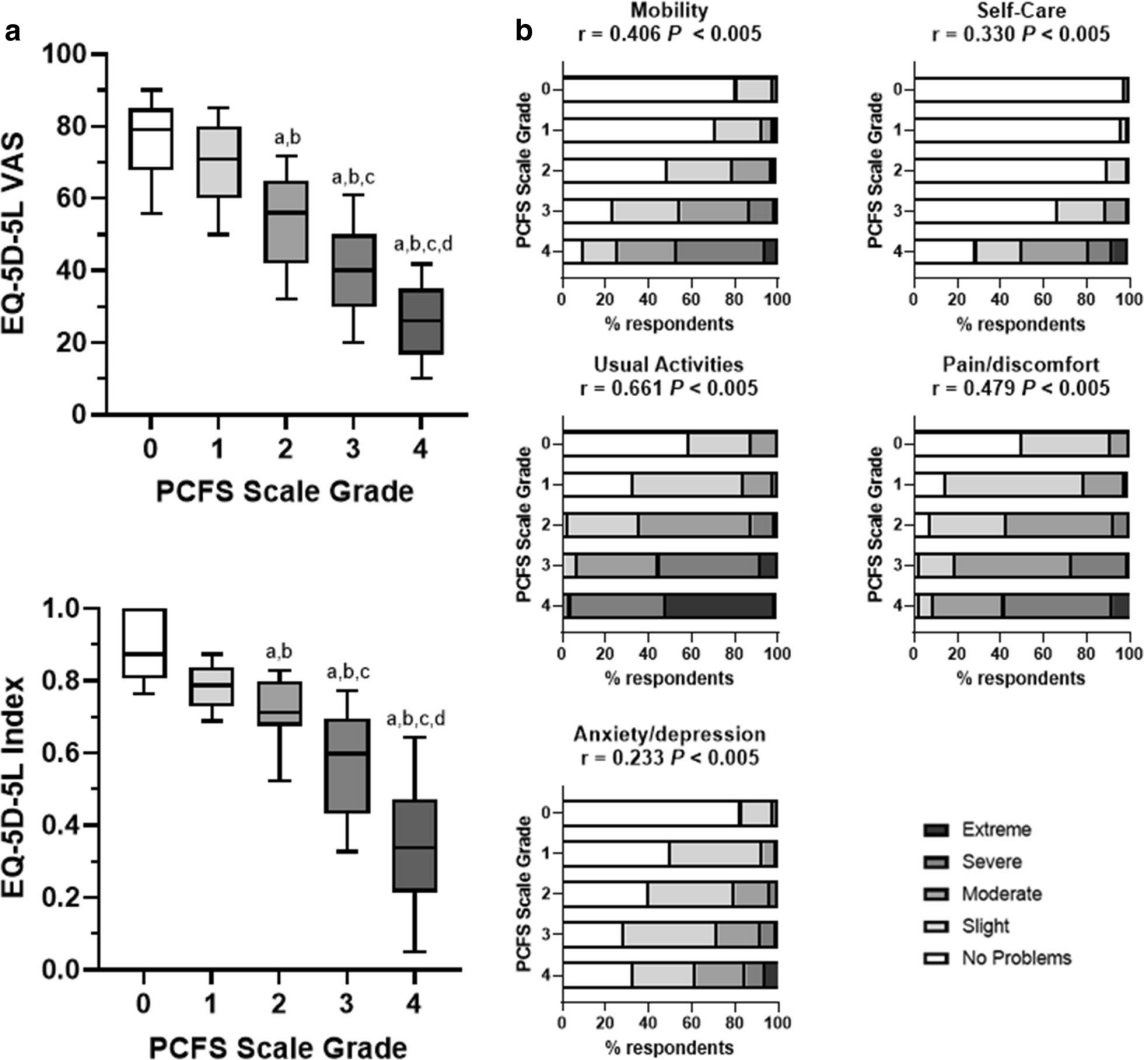
**A** Comparisons of HrQoL of life between subjects with COVID-19 stratified according to the level of impairment in functional status. **a** *P* < 0.05 compared with Grade 0; **b** *P* < 0.05* compared with Grade 1;*** c** *P* < 0.05 *compared with Grade 2;*** d** *P* < 0.05* compared with Grade 3.* Figure displays the median as central line, interquartile range as the limits of the box and 10 and 90 percentiles as whiskers. **B** Associations between level of impairment in functional status and different domains of HrQoL

HrQoL was comparable between subjects with negligible functional limitations and no functional limitations, while all other pairwise comparisons including the subjects with slight, moderate and severe functional limitations showed worsening of HrQoL for increasing categories of the PCFS Scale (Fig. 2a). We found weak-to-strong associations between functional status and all domains of HrQoL (*r*: 0.233–0.661; *P* < 0.01) (Fig. 2b). Notably, the strongest association found was with the ‘usual activities’ domain of the 5-level EQ-5D questionnaire.

**Fig. 3:**
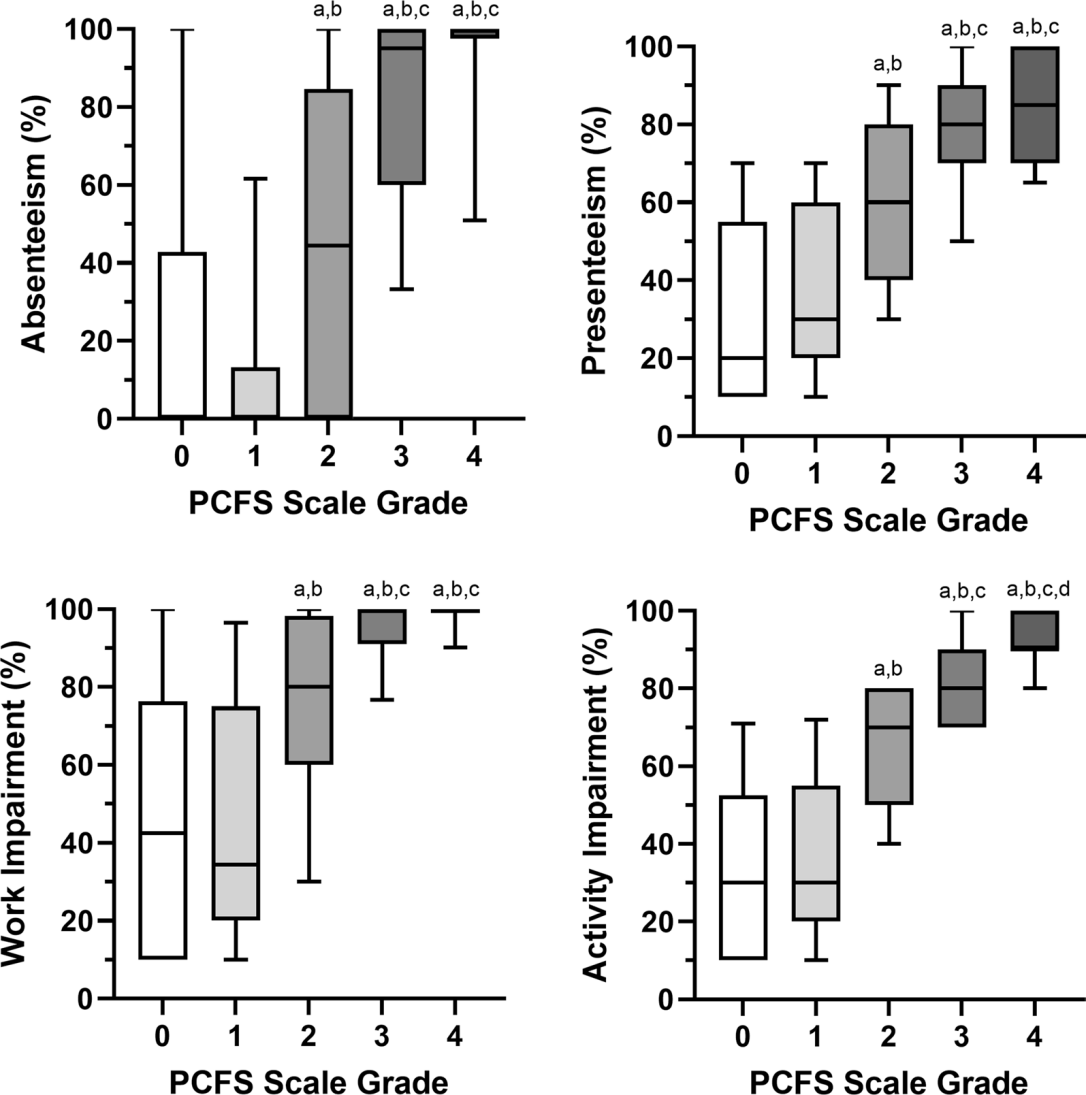
Comparisons of work productivity and activity impairment between subjects with COVID-19 stratified according to the level of impairment in functional status. **a** *P* < 0.05 compared with Grade 0; **b** * P* < 0.05* compared with Grade 1;* **c*** P* < 0.05* compared with Grade 2;*** d** *P* < 0.05* compared with Grade 3.* Figure displays the median as central line, interquartile range as the limits of the box and 10 and 90 percentiles as whiskers

Figure 3 displays the comparison of the level of impairment in work and usual activities as assessed by the WPAI questionnaire. Absenteeism, presenteeism and work impairment were different considering all pairwise comparisons with exception of the comparison between subjects with no and negligible functional limitations and between subjects with moderate and severe functional limitations. Activity impairment increased gradually according to the decrease in functional status.

## Discussion

This is the first study to describe the PCFS Scale in a large sample of adult hospitalized and non-hospitalized subjects with confirmed and presumed COVID-19. The present results confirm the hypothesis that the PCFS Scale can be used to measure the impact of symptoms on the functional status of subjects after COVID-19, especially in slight to severe categories. We found gradual differences in the number and intensity of symptoms, reduction in HrQoL and impairment in work and usual activities between subjects with slight, moderate and severe functional limitation status (≥ grade 2) while no differences between subjects with no functional limitation (grade 0) and negligible functional limitation (grade 1) were found when comparing these outcomes, although a lack of power to detect small but meaningful differences among the latter groups might have affected these results.

The majority of the included subjects was not formally tested during the infection (78%), whereas a minority was hospitalized with confirmed diagnosis of COVID-19 (5%). These findings are expected, as early data showed that the proportion of critical cases of COVID-19 is 5% [2] and the number of undocumented infections have been estimated at 86% of all infections [3]. When proposing the PCFS Scale, Klok and colleagues underlines the relevance of the assessment of functional status in the weeks and months following acute care of COVID-19 subjects [8]. Our findings describe that, despite the inclusion of a middle aged sample of subjects without multiple comorbidities and with diagnosis of ‘mild’ COVID-19 (*i.e.* non-hospitalized during the infection), the majority of them (85%) report slight-to-moderate functional limitations during course of COVID-19, 3 months after the onset of symptoms, supporting the existence of a ‘post-COVID-19 syndrome’ [14] which may be a target for deciding on referral of non-hospitalized subjects to expert (outpatient) clinics, facilitate selection of subjects who may benefit from rehabilitation programs as well as measuring the efficacy of such programs.

Of note, one of the strong points of the scale is that it intentionally does not specify why subjects are functionally impaired, i.e. if the impairment is caused by dyspnoea, fatigue, pain, anxiety or otherwise. This is crucial for the scale to be useful in a condition that may cause symptoms in many organ systems. However, it does not rule out the fact that the reported functional limitations are (partly) pre-existent. This is no issue when the scale is measured repeatedly or used as outcome measure in studies comparing two or more groups, especially in randomized controlled trials. The manual to the scale describes the possibility to assess pre-COVID-19 functional status, which would allow for determination of the causality of functional impairment in observational studies [10]. Importantly, since the aim of the current study was to assess the associations between measures of symptoms intensity, HrQoL, impairment in work and usual activities due to health and the PCFS Scale, these considerations do not pose an issue for the correct interpretation of the presented data, nor does the fact that COVID-19 was not confirmed by objective testing in all subjects.

As most of the included subjects in our study were highly symptomatic and the PCFS Scale has shown to be associated with number and intensity of symptoms, the groups of subjects stratified as presenting no functional limitations (grade 0) and negligible functional limitations (grade 1) were relatively small (3% and 8% of total sample, respectively) likely due to a selection bias arising from the self-selection nature of the data. Also, the lack of statistically significant differences between these groups could be a direct consequence of this limited number of subjects. It is also possible that there is, in fact, not a clinically relevant difference in outcomes between these two grades of the PCFS Scale, future studies with inclusion of less symptomatic subjects are necessary to investigate this issue. After all, both a scale grade of 0 and 1 indicate no functional limitations. The clinical relevance between the 2 grades is possibly to differentiate full recovery (grade 0) from incomplete recovery and persistent symptoms (grade 1).

The groups of subjects stratified as presenting slight and moderate functional limitations (grade 2 and 3) present similar baseline characteristics, however with a gradual increase in number/intensity of symptoms, reduction in HrQoL and impairment in work and usual activities. Despite presenting significantly lower BMI, the relatively small group of subjects with severe functional limitations (grade 4) was found to be highly symptomatic and presenting worse HrQoL. This could be partially explained by the higher proportion of subjects with multimorbidity included in this group, since previous studies have found that multimorbidity is an independent factor associated with HrQoL [24, 25]. Furthermore, this group of subjects also presented a higher proportion of subjects with other factors that are associated with poorer functional status such as living alone and presenting a ‘symptom-based’ COVID-19 diagnosis.

The main limitation of the present study is the inclusion of a self-selected sample of subjects with confirmed and presumed COVID-19 who are still symptomatic after the infection, it is possible that the frequency and intensity of symptoms reported by non-hospitalized subjects is overestimated due to selection bias and due to the inclusion of a relatively high proportion of women, who usually report more symptoms than men [26]. Moreover, if COVID-19 ICU survivors and other hospitalized subjects would have been included, the distribution of the PCFS could have shifted towards more symptoms and functional impairment. Future studies should investigate the distribution of the scale grades in less selected cohorts of COVID-19 subjects, as well as confirm the identified associations in other samples including a higher proportion of men, less symptomatic subjects and in sequential assessment of the scale during the course of disease (e.g. at the time of hospital discharge, more than 3 months after the onset of symptoms). In addition, we did not include any objective measure of physical functioning that could be related to functional status, however the strong association with the domain ‘usual activities’ of the EQ-5D-5L and WPAI and the ability to discriminate subjects with higher number/intensity of symptoms demonstrate the construct validity of the PCFS Scale.

## Conclusion

In conclusion, we demonstrated the construct validity of the PCFS Scale in highly-symptomatic adult subjects with confirmed or presumed COVID-19 3 months after the onset of symptoms. The fact that the PCFS Scale can be easily used is a major advantage of the scale, potentially facilitating its widespread implementation. We propose that this tool could be used to discriminate between subjects with higher number and intensity of symptoms which is related with reduced HrQoL and impairment in work and usual activities. Therefore, the scale could be used to guide follow-up procedures such as referral to expert (outpatient) clinics or rehabilitation programs. Also, this shows that the PCFS Scale could be considered as a main outcome in clinical trials as well as observational studies, as it captures symptom intensity and HrQoL in one meaningful scale. The construct validity is a first step towards the validation of the PCFS Scale and more studies assessing other measurement properties are recommended.

## Supplementary Information

**Additional file 1**. Online Supplement.

## Data Availability

The datasets used and analysed during the current study are available from the corresponding author on reasonable request.

## Abbreviations

BMI: Body mass index
COVID-19: Corona virus disease 2019
CT: Computed tomography
EQ-5D-5L: 5-Level version of the EQ-5D questionnaire
HR-PRO: Health-related patient-reported outcomes
HrQoL: Health-related quality of life
ICU: Intensive care unit
PCFS: Post-COVID-19 Functional Status
RT-PCR: Reverse transcription polymerase chain reaction
SARS: Severe acute respiratory syndrome
SARS-CoV-2: Severe acute respiratory syndrome coronavirus 2
VTE: Venous thromboembolism
VAS: Visual analogue scale
WPAI: Work Productivity and Activity Impairment

## References

[1] WHO. Weekly operational update on COVID-19. https://www.who.int/publications/m/item/weekly-epidemiological-update---12-january-2021. Accessed 17 Jan 2021.

[2] Wu Z, McGoogan JM (2020). Characteristics of and important lessons from the coronavirus disease 2019 (COVID-19) outbreak in China. JAMA.

[3] Li R, Pei S, Chen B, Song Y, Zhang T, Yang W (2020). Substantial undocumented infection facilitates the rapid dissemination of novel coronavirus (SARS-CoV-2). Science.

[4] Flaxman S, Mishra S, Gandy A, Unwin HJT, Mellan TA, Coupland H (2020). Estimating the effects of non-pharmaceutical interventions on COVID-19 in Europe. Nature.

[5] Tansey CM, Louie M, Loeb M, Muller MP, Jager J, Cameron JI (2007). One-year outcomes and health care utilization in survivors of severe acute respiratory syndrome. Arch Intern Med.

[6] Hui DS, Joynt GM, Wong KT, Gomersall CD, Li TS, Antonio G (2005). Impact of severe acute respiratory syndrome (SARS) on pulmonary function, functional capacity and quality of life in a cohort of survivors. Thorax.

[7] Ngai JC, Ko FW, Ng SS, To K-W, Tong M, Hui DS (2010). The long-term impact of severe acute respiratory syndrome on pulmonary function, exercise capacity and health status. Respirology.

[8] Klok FA, Boon GJAM, Barco S, Endres M, Geelhoed JJM, Knauss S (2020). The Post-COVID-19 Functional Status (PCFS) Scale: a tool to measure functional status over time after COVID-19. Eur Respir J.

[9] Klok FA, Barco S, Siegerink B (2019). Measuring functional limitations after venous thromboembolism: a call to action. Thromb Res.

[10] Boon GJAM, Barco S, Bertoletti L, Ghanima W, Huisman MV, Kahn SR (2020). Measuring functional limitations after venous thromboembolism: optimization of the Post-VTE Functional Status (PVFS) Scale. Thromb Res.

[11] Klok FA, Kruip MJHA, van der Meer NJM, Arbous MS, Gommers D, Kant KM (2020). Confirmation of the high cumulative incidence of thrombotic complications in critically ill ICU patients with COVID-19: an updated analysis. Thromb Res.

[12] Public Facebook Group: Corona patiënten met langdurige klachten (Vlaanderen). 2020. https://www.facebook.com/groups/241043323639334.

[13] Public Facebook Group: Corona ervaringen en langdurige klachten (Nederland). 2020. https://www.facebook.com/groups/236723204035929.

[14] Goërtz YMJ, van Herck M, Delbressine JM, Vaes AW, Meys R, Machado FVC (2020). Persistent symptoms 3 months after a SARS-CoV-2 infection: the post-COVID-19 syndrome?. ERJ Open Res.

[15] Vaes AW, Machado FVC, Meys R, Delbressine JM, Goërtz YMJ, van Herck M (2020). Care dependency in non-hospitalized patients with COVID-19. J Clin Med.

[16] Meys R, Delbressine JM, Goërtz YMJ, Vaes AW, Machado FVC, van Herck M (2020). Generic and respiratory—specific quality of life in non-hospitalized patients with COVID. J Clin Med.

[17] Mokkink LB, Terwee CB, Patrick DL, Alonso J, Stratford PW, Knol DL (2010). The COSMIN study reached international consensus on taxonomy, terminology, and definitions of measurement properties for health-related patient-reported outcomes. J Clin Epidemiol.

[18] Hui D, Bruera E (2017). The Edmonton symptom assessment system 25 years later: past, present, and future developments. J Pain Symptom Manag.

[19] Zeng L, Zhang L, Culleton S, Jon F, Holden L, Kwong J (2011). Edmonton symptom assessment scale as a prognosticative indicator in patients with advanced cancer. J Palliat Med.

[20] EuroQol Research Foundation. EQ-5D-5L User Guide, 2019. https://euroqol.org/publications/user-guides.

[21] Versteegh MM, Vermeulen KM, Evers SMAA, de Wit GA, Prenger R, Stolk EA (2016). Dutch tariff for the five-level version of EQ-5D. Value Health.

[22] Reilly MC, Zbrozek AS, Dukes EM (1993). The validity and reproducibility of a work productivity and activity impairment instrument. Pharmacoeconomics.

[23] Miller PSJ, Hill H, Andersson FL (2016). Nocturia work productivity and activity impairment compared with other common chronic diseases. Pharmacoeconomics.

[24] Fortin M, Lapointe L, Hudon C, Vanasse A, Ntetu AL, Maltais D (2004). Multimorbidity and quality of life in primary care: a systematic review. Health Qual Life Outcomes.

[25] Fortin M, Bravo G, Hudon C, Lapointe L, Almirall J, Dubois M-F (2006). Relationship between multimorbidity and health-related quality of life of patients in primary care. Qual Life Res.

[26] Bardel A, Wallander M-A, Wallman T, Rosengren A, Johansson S, Eriksson H (2019). Age and sex related self-reported symptoms in a general population across 30 years: patterns of reporting and secular trend. PLoS ONE.

