# Supplementary material for: Construct validity of the Post-COVID-19 Functional Status Scale in adult subjects with COVID-19
Source: Health Qual Life Outcomes. 2021 Feb 3;19:40. doi: 10.1186/s12955-021-01691-2 (PMC7856622; doi:10.1186/s12955-021-01691-2)

**ONLINE SUPPLEMENT**

**Title:** Construct validity of the Post-COVID-19 Functional Status Scale in adult subjects with COVID-19

**Authors’ full names:** Felipe V. C. Machado^1,2,3,4^, Roy Meys^1,2,3^, Jeannet M. Delbressine^1^, Anouk W. Vaes^1^, Yvonne M. J. Goertz^1,2,3^, Maarten Van Herck^1,2,3,5^, Sarah Houben-Wilke^1^, Gudula J.A.M. Boon^6^, Stefano Barco^7,8^, Chris Burtin^5^, Alex van ’t Hul^9^, Rein Posthuma^1,2,3^, Frits M.E. Franssen^1,2,3^, Yvonne Spies^10^, Herman Vijlbrief^10^, Fabio Pitta^4^, Spencer A. Rezek^11^, Daisy J.A. Janssen^1,12^, Bob Siegerink^13,14^, Frederikus A. Klok^6^, Martijn A. Spruit^1,2,3,5^

**Authors’ affiliations:**

^1^Department of Research and Development, CIRO+, Horn, The Netherlands.

^2^NUTRIM School of Nutrition and Translational Research in Metabolism, Maastricht, The Netherlands.

^3^Department of Respiratory Medicine, Maastricht University Medical Center (MUMC+), Maastricht, The Netherlands.

^4^Laboratory of Research in Respiratory Physiotherapy (LFIP), Department of Physiotherapy, State University of Londrina (UEL), Londrina, Brazil.

^5^REVAL Rehabilitation Research Center, BIOMED Biomedical Research Institute, Faculty of Rehabilitation Sciences, Hasselt University, Diepenbeek, Belgium.

^6^Department of Medicine – Thrombosis and Hemostasis, Leiden University Medical Center, Leiden, The Netherlands.

^7^Center for Thrombosis and Haemostasis (CTH), University Medical Center of the Johannes Gutenberg University Mainz, Mainz, Germany.

^8^Clinic of Angiology, University Hospital Zurich, Zurich, Switzerland.

^9^Department of Pulmonary Diseases, Radboud University Medical Center, 6525 GA, Nijmegen, The Netherlands.

^10^Lung Foundation Netherlands, Amersfoort, The Netherlands.

^11^Institute of Therapies and Rehabilitation, Kantonsspital Winterthur, Winterthur, Switzerland.

^12^Department of Health Services Research, Care and Public Health Research Institute, Faculty of Health, Medicine and Life Sciences, Maastricht University, Maastricht, The Netherlands.

^13^Center for Stroke research Berlin, Charité Universitätsmedizin Berlin, Berlin, Germany.

^14^Department of Clinical Epidemiology, Leiden University Medical Center, Leiden, The Netherlands.

**Corresponding author:**

Felipe V. C. Machado

Dept. of Research and Development, Ciro

PO Box 4080

6080 AB HAELEN

The Netherlands

T +31 (0)475 587 600

felipemachado@ciro-horn.nl

| **Pre-existing comorbidities** |  | **Post-COVID-19 Functional Status Scale** | | | | |
| --- | --- | --- | --- | --- | --- | --- |
|  | **All Sample** | **Grade = 0** | **Grade = 1** | **Grade = 2** | **Grade = 3** | **Grade = 4** |
|  | **(n=1939)** | **(n=58)** | **(n=157)** | **(n=643)** | **(n=1011)** | **(n=70)** |
| Anxiety, n (%) | 46 (2.4) | 1(1.7) | 3(1.9) | 8 (1.2) | 28 (2.8) | 6 (8.6) |
| Arthrosis, rheumatoid arthritis, n (%) | 101 (5.2) | 2(3.4) | 9(5.7) | 29(4.5) | 55(5.4) | 6(8.6) |
| Burn-out, n (%) | 58(3.0) | 0(0) | 3(1.9) | 17(2.6) | 33(3.3) | 5(7.1) |
| Cancer, n (%) | 12(0.6) | 1(1.7) | 0(0) | 7(1.1) | 4(0.4) | 0(0) |
| Depression, n (%) | 63(3.3) | 0(0) | 4(2.5) | 17(2.6) | 40(4) | 3(3.4) |
| Diabetes, n (%) | 27(1.4) | 0(0) | 2(1.3) | 7(1.1) | 14(1.4) | 4(5.7) |
| Heart diseases, n (%) | 52(2.7) | 2(3.4) | 1(0.6) | 17(2.6) | 26(2.6) | 6(8.6) |
| Hypertension, n (%) | 137(7.1) | 4(6.9) | 14(8.9) | 49(7.6) | 64(6.3) | 6(8.6) |
| Kidney diseases, n (%) | 7(0.4) | 0(0) | 1(0.6) | 3(0.5) | 2(0.2) | 1(1.4) |
| Lung diseases, n (%) | 217(11.2) | 4(6.9) | 16(10.2) | 60(9.3) | 124(12.3) | 13(18.6) |
| Migraine, n (%) | 67(3.5) | 3(5.2) | 4(2.5) | 23(3.6) | 31(3.1) | 6(8.6) |
| Obesity, n (%) | 27(1.4) | 1(1.7) | 3(1.9) | 8(1.2) | 14(1.4) | 1(1.4) |
| Osteoporosis, n (%) | 15(0.8) | 0(0) | 0(0) | 3(0.5) | 10(1.0) | 2(2.9) |
| Other, n (%) | 302(15.6) | 8(13.8) | 13(8.3) | 94(14.6) | 166(16.4) | 21(30.0) |
| Parkinson’s disease , n (%) | 0(0) | 0(0) | 0(0) | 0(0) | 0(0) | 0(0) |
| Stroke, n (%) | 6(0.3) | 0(0) | 0(0) | 4(0.6) | 2(0.2) | 0(0) |

**Online Table 1.** Pre-existing comorbidities of the subjects with COVID-19 stratified according to the level of impairment in functional status.

**Online Figure 1.** Associations between baseline characteristics and the likelihood of being classified with a higher level of impairment in functional status assessed by the PCFS Scale. OR: Odds ratio [95% confidence intervals] reported. OB: obese; OW: overweight; NW: normal weight; UW: underweight


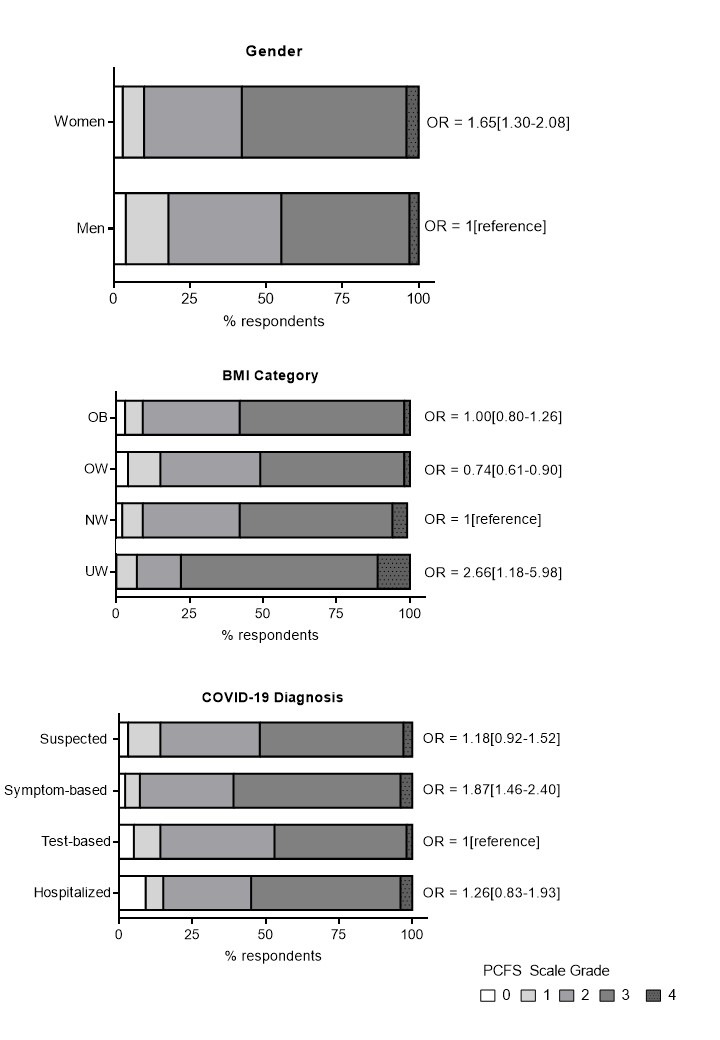


**Online Figure 1 (continued).**


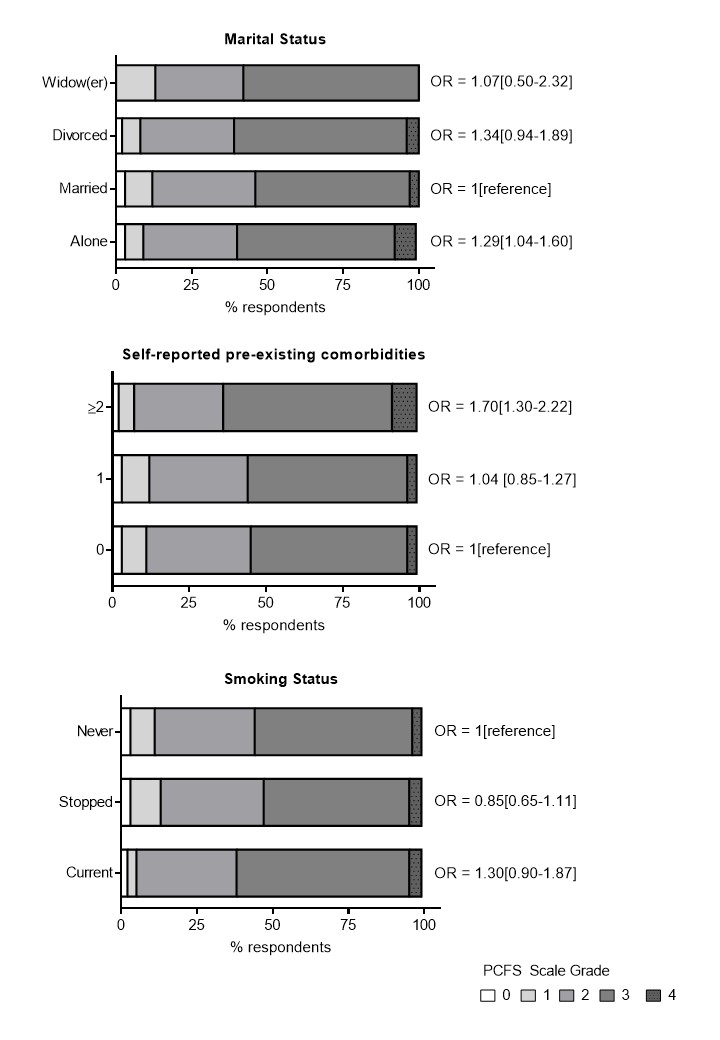

Supplement: Supplementary file 1 — Additional file 1. Online Supplement. [file 12955_2021_1691_MOESM1_ESM.docx]
